# A case report: Neuroimaging in an atypical presentation of Parkinson’s disease

**DOI:** 10.4102/sajpsychiatry.v32i0.2522

**Published:** 2026-03-12

**Authors:** Sibusiso N. F. Sotobe Mose, Karishma Lowton

**Affiliations:** 1 Department of Health, Faculty of Psychiatry, University of the Witwatersrand, Johannesburg, South Africa

**Keywords:** Idiopathic Parkinson’s disease, atypical parkinsonism, Parkinson’s disease dementia, functional imaging, diagnostic evaluation

## Abstract

**Introduction:**

Parkinson’s disease (PD) is the second leading neurodegenerative disorder in the world. The diagnosis of idiopathic Parkinson’s disease (IPD) is mainly through clinical presentation of motor and non-motor symptoms. Motor symptoms include tremor, bradykinesia and rigidity. Atypical parkinsonism may pose a challenge in diagnosing IPD. Functional neuroimaging can assist in diagnosing IPD and in differentiating it from atypical parkinsonism due to other neurodegenerative aetiologies.

**Patient presentation:**

We present a case report of a patient with atypical parkinsonism and mild neurocognitive disorder.

**Management and outcome:**

Neuroimaging revealed IPD, distinguishing it from other causes of neurocognitive disorders. The patient received multidisciplinary team (MDT) input and appropriate medication, including a fixed combination of carbidopa and levodopa, rivastigmine, venlafaxine and quetiapine with improvement of his symptomatology.

**Conclusion:**

Neuroimaging assisted in establishing the diagnosis and guiding treatment.

**Contribution:**

Although evidence and studies are needed for definitive use in clinical practice, there is supportive evidence to suggest the diagnostic utility of these modalities in parkinsonian syndromes.

## Introduction and literature review

Differentiating idiopathic Parkinson’s disease (IPD) from other parkinsonian diseases, including Lewy body dementia (LBD), multiple system atrophy (MSA), progressive supranuclear palsy (PSP) and corticobasal degeneration (CBD), is often challenging.^1,2,3,4^ Furthermore, there may be challenges in distinguishing LBD and Parkinson’s disease dementia (PDD).^3^ Overlaps in these disorders include neuropathology^3,5^ and clinical presentation, which include parkinsonism, cognitive fallout, visual hallucinations, sleep disturbances and autonomic dysfunction.^3,6^ Neurocognitive symptoms preceding parkinsonism by up to a year, along with a poor response to levodopa trials, may aid in distinguishing LBD from IPD.^1,3,5,6,7^

Neuroimaging used in IPD includes magnetic resonance imaging (MRI), single photon emission computed tomography (SPECT), positron emission tomography (PET) and metaiodobenzylguanidine (MIBG) scans.^3,4,5,8,9^ Magnetic resonance imaging helps distinguish structural changes associated with other parkinsonian diseases; for example, changes in putamen signal suggest MSA and ischaemic changes suggest vascular pathology.^4^ Positron emission tomography scans, although helpful, lack specificity as a biomarker for LBD and IPD.^3,4,5^ Although costly, dopamine transporter (DAT) scan (SPECT or PET) and MIBG scans are more specific and diagnostic for both LBD and IPD.^3,4,5^

Dopamine transporter (DAT) scans have demonstrated high sensitivity (79%–87%) and specificity (80%–100%) in distinguishing IPD from secondary causes of parkinsonism, such as vascular or drug-induced parkinsonism.^4^ In LBD, a DAT scan may be normal or show diffuse reduction in striatal uptake.^3,10^ In IPD, reduced DAT uptake is more prominent in the hemisphere contralateral to the presenting symptoms, typically displaying a comma-shaped pattern involving the anterior caudate and posterior putamen.^3^

The case report illustrates the benefit of diagnostic functional imaging in a patient with atypical parkinsonism, originally referred with a possible LBD diagnosis.

## Ethical considerations

Ethical clearance to conduct this study was obtained from the Human Research Ethics Committee (Medical) at the University of the Witwatersrand (No. M241089). Written informed consent was obtained from the patient.

## Patient presentation

### Case report

Mr H is a 72-year-old man who presented to Charlotte Maxeke Johannesburg Academic Hospital neuropsychiatry clinic on 29 August 2022. He was referred by the neurology department for assessment, diagnostic clarity, and management thereof. He was diagnosed with a cerebrovascular accident (CVA) in January 2019 because of paradoxical cerebral embolus from a congenital patent foramen ovale that required transoesophageal surgery and anticoagulant therapy till 2022. Three months after his 2019 CVA, he developed subtle, insidiously progressive subcortical cognitive deficits (attention, working memory and word retrieval), along with a resting tremor of the right upper limb. His cognitive symptoms and tremor worsened in the year prior to presentation at the neuropsychiatry clinic. He cannot recall which symptom cluster emerged first; however, both the cognitive and motor symptoms began within the same year following the CVA.

Other associated symptoms included significant rapid eye movement (REM) sleep behaviour disorder (RBD), resulting in injury to his wife, with onset in February 2021. He reported the onset of vivid, detailed, colourful visual hallucinations in May 2022, with urinary urgency reported in August 2022, followed by constipation in November 2022. Although he remained independent in all activities of daily living, he reported increased cognitive and physical effort required to maintain this level of functioning.

His past psychiatry history revealed diagnoses of major depressive disorder and generalised anxiety disorder at 62 years, which remitted on fluoxetine 20 mg. This was changed to citalopram 20 mg and quetiapine 200 mg in 2021 at Helen Joseph Hospital following a relapse of neurobehavioural symptoms. Upon review, he reported ongoing generalised anxiety as a result of worsening cognitive symptoms and financial difficulties.

He was diagnosed with dyslipidaemia, rheumatoid arthritis and a previous mild coronavirus disease 2019 (COVID-19) infection in December 2020. His mother was diagnosed in her 70s with major neurocognitive disorder due to possible Alzheimer’s disease.

The following differential diagnoses were considered:

- Mild neurocognitive disorder due to possible Lewy body disease.
- Mild neurocognitive disorder due to possible vascular disease.
- Mild neurocognitive disorder due to possible idiopathic Parkinson’s disease.

**FIGURE 1:**
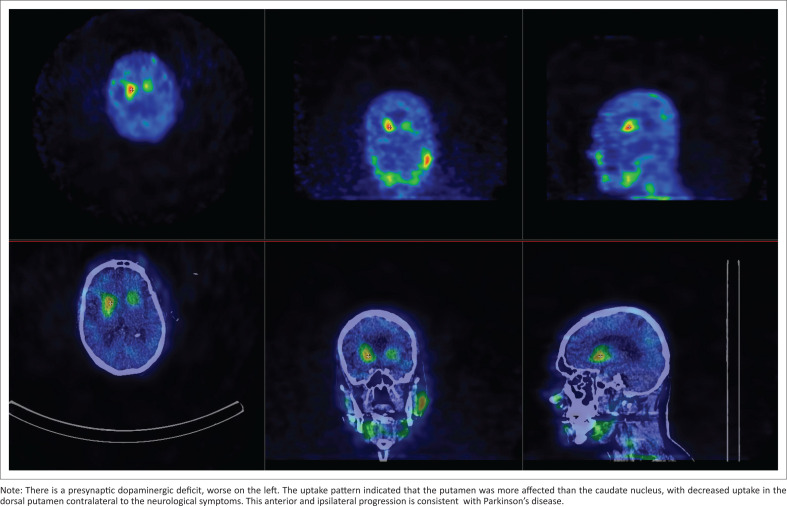
Brain Single Photon Emission Computed Tomography (SPECT).

His MRI brain (23 January 2019) showed microemboli involving the posterior circulation, with features of microvascular ischaemia. Positron emission tomography imaging (14 April 2023) was reported as normal and inconclusive, which did not align with the patient’s clinical presentation. A DAT scan was requested to clarify the underlying aetiology of the patient’s neurocognitive disorder in the context of parkinsonism. The DAT scan (02 September 2023) revealed a presynaptic striatal dopaminergic deficit (worse on the left), suggestive of IPD. This was not in keeping with the scintigraphy features of LBD or vascular causes, where diffuse, significantly striatal binding is noted. See Figure 1.

He was managed by the multidisciplinary team (MDT), including occupational therapy and neuropsychology. Citalopram was discontinued, venlafaxine was initiated and titrated to 150 mg daily, and quetiapine was increased to 250 mg, with significant symptomatic improvement. Mr H was also started on a fixed combination of carbidopa and levodopa, as well as rivastigmine. His hallucinations remitted, and RBD symptoms improved. Education regarding safety precautions and behavioural management of RBD was provided to Mr H and his wife. He was ambulant without assistive devices that were previously required and displayed no active neurobehavioural symptoms at his last follow-up appointment.

## Conclusion

Functional imaging with appropriate tracers is a valuable diagnostic biomarker for distinguishing the aetiologies of atypical parkinsonism associated with neurocognitive disorders. These modalities should be advocated, particularly in complex cases where multiple conditions may contribute to the clinical presentation.
